# A Rare Incidental Finding of a Foreign Body in the Nasopharynx during Adenotonsillectomy

**DOI:** 10.1155/2018/8361806

**Published:** 2018-03-28

**Authors:** Waleed M. Alshehri, Bandar Al-Qahtani

**Affiliations:** Otolaryngology Department, King Saud Medical City, Riyadh 12746, Saudi Arabia

## Abstract

Diverse foreign bodies may become lodged in the aerodigestive tract, and the discovery of such foreign bodies is an expected scenario for health-care practitioners. The foreign body insertion may be accidental or deliberate, and the object may be organic or inorganic. Most accidental foreign body aspirations occur in children, and some such cases are potential threats that go unnoticed. Very few cases of foreign bodies in the nasopharynx have been reported. Herein, we describe an unusual case in which a foreign body in a child's nasopharynx went unnoticed for 1 year and was detected intraoperatively.

## 1. Introduction

The peer-reviewed literature contains reports of over 12,000 pediatric cases of aspirated foreign bodies that required bronchoscopic removal [1]. Clinical suspicion is initially based on the patient's history, the reported symptoms, and an objective chest evaluation [2, 3] incorporating X-ray imaging and airway fluoroscopy [4–6]. Bronchoscopy is used to establish a definitive diagnosis [7, 8], and a rigid bronchoscope is the ideal tool for removal [9–11].

Multiple retrospective series studies of pediatric foreign body aspiration have helped in the epidemiological characterization of this condition [1, 9, 12–18]. The lodgment sites of aspirated foreign bodies are diverse. Eren et al. [14] reviewed over 1,000 pediatric cases of foreign body aspiration and reported that the bronchoscopically detected lodgment sites included the larynx (3%), trachea (13%), right main bronchus or distal branch (60%), and left main bronchus or distal branch (23%). However, the nasopharynx is a rare lodgment site for foreign bodies and hardly receives a passing mention even in standard comprehensive textbooks [19]. Herein, we describe a rare pediatric case of nasopharyngeal lodgment of an aspirated foreign body.

## 2. Case Presentation

**Figure 1:**
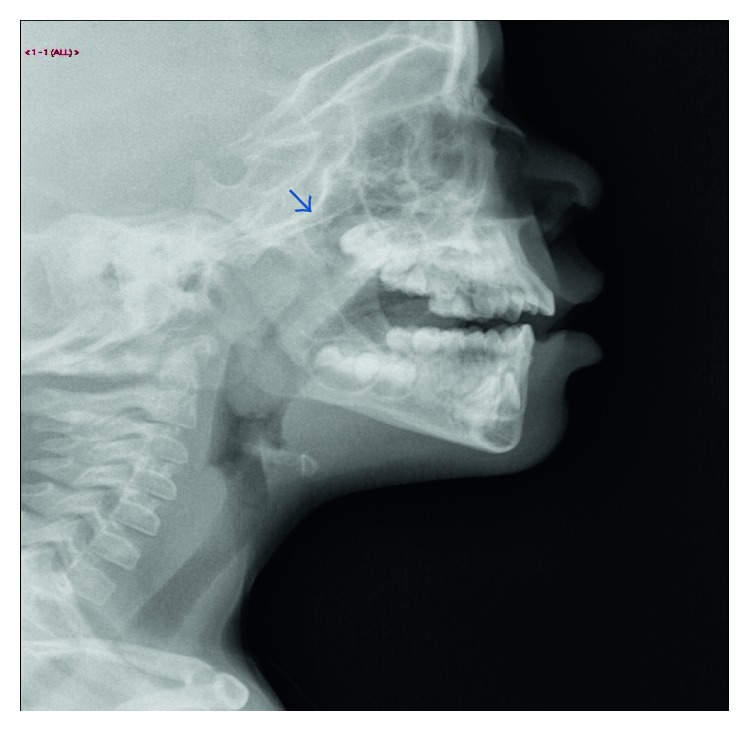
Soft tissue X-ray image showing adenoid enlargement.

A 4-year-old girl was admitted to undergo an elective adenotonsillectomy as a day surgery patient. The child had been experiencing recurrent adenotonsillitis and snoring. She had unremarkable nose and ear examination results and grade 2 tonsils according to the Brodsky scale. She underwent a routine preoperative assessment in which all laboratory test results were normal. However, an adenoid X-ray examination showed posterior nasopharyngeal soft tissue that was indenting and narrowing the nasopharyngeal air column (Figure 1), which suggested adenoid hypertrophy. We observed a faint soft tissue structure (19 mm × 13 mm) in the oropharynx. The soft tissue shadows of the epiglottis and the cervical spine, and the prevertebral soft tissue shadow, all looked normal. When we examined the nasopharynx with a mirror to visualize the adenoid tissue, we observed a foreign body in the choanae.

**Figure 2:**
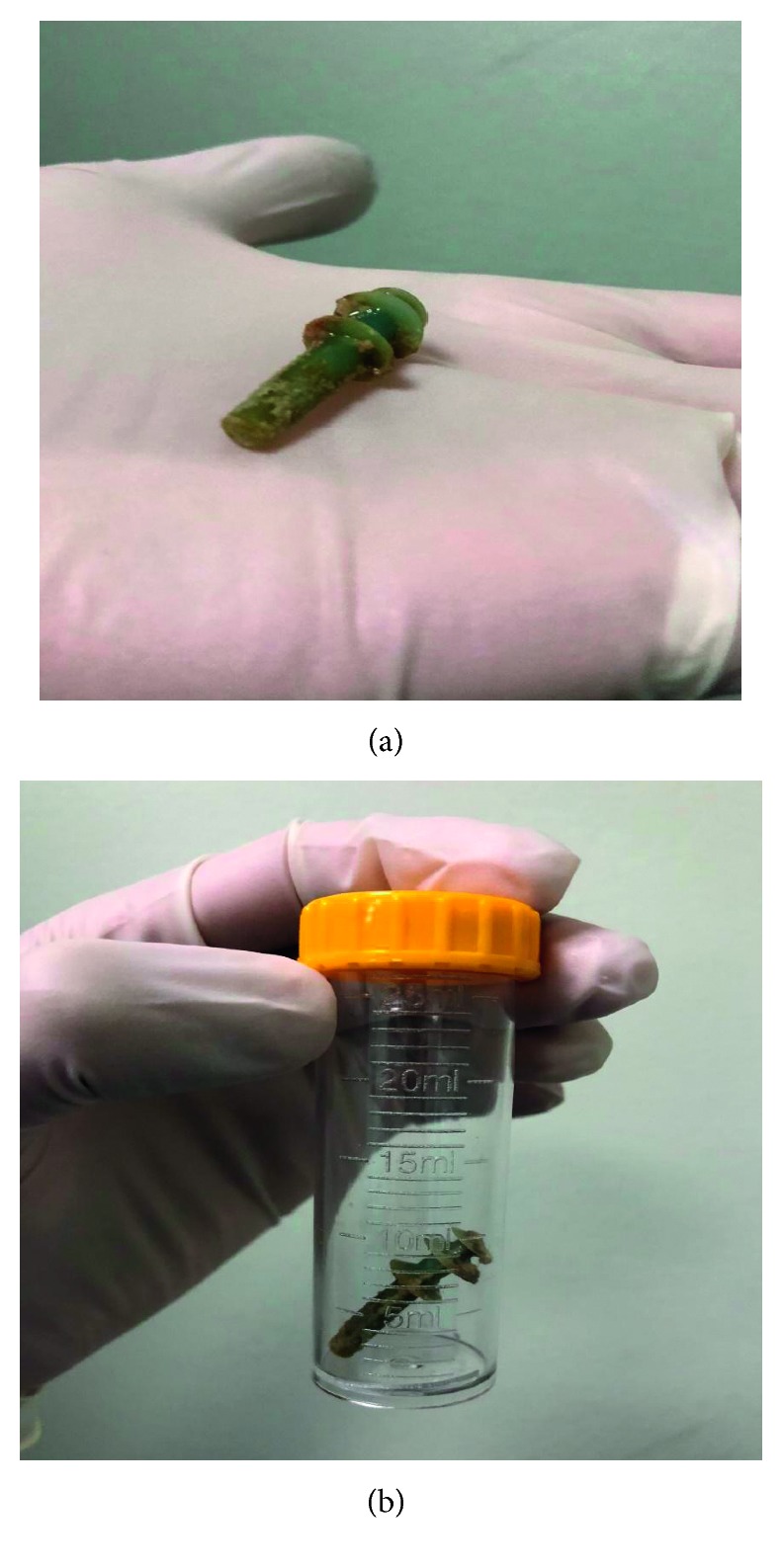
Foreign body after removal.

We removed the foreign body with curved artery forceps. We found that the specimen was a rubber swimming earplug for adults (Figure 2). The adenotonsillectomy was continued as planned with no intraoperative complications, and the patient was discharged later that day.

After the operation, we asked the patient's mother about the foreign body, and she mentioned that her daughter had experienced a brief choking episode 1 year earlier. She had taken the child to a pediatric emergency room unaware that she had aspirated a foreign body. The emergency room physicians had obtained an X-ray image but observed no otolaryngologic irregularities. The child had been discharged later the same day in good condition.

The child's mother provided consent for the publication of this case report.

## 3. Discussion

Children who aspirate foreign bodies present with signs and symptoms that are mostly nonspecific, and in cases of unwitnessed aspiration, the diagnosis can be delayed, which increases morbidity and the likelihood of chronicity [20]. Children younger than 36 months have a normal developmental curiosity and independence associated with reduced parental supervision. Altogether, these factors place them at an increased risk of foreign body aspirations; this age group has been shown to account for approximately 75% of reported cases [21, 22]. A positive suggestive history of aspiration is less likely in long-standing cases of otolaryngologic irregularities, with the likelihood of foreign body aspiration reportedly ranging from 53% to 77% [23, 24]. Despite the voluminous literature addressing pediatric foreign body aspiration, few studies have examined the clinical characteristics of cases involving delayed presentation [25, 26].

The nasopharynx is an uncommon lodgment site for foreign bodies. This was confirmed by our literature search, which revealed very few reports of nasopharyngeal foreign body lodgment. The reported cases involved small objects such as a ring [27], a tooth [28], a leech [29], and even a fish [30]. Nasopharyngeal foreign body lodgment can occur in many scenarios, such as dislocation of a foreign body from the nasal cavity during extraction attempts, upward migration from the pharynx or esophagus after forceful pressure due to vomiting or coughing, traumatic penetration, or iatrogenic causes [30]. The clinical presentation of nasopharyngeal foreign body aspiration may mimic other common pediatric conditions such as adenoid hypertrophy or rhinosinusitis [31]. The most common patient complaints include bilateral purulent rhinorrhea and nasal obstruction. Epistaxis, recurrent rhinosinusitis, halitosis, and otitis media with or without effusion may also occur in cases of prolonged nasopharyngeal lodgment [30].

## 4. Conclusion

Nasopharyngeal foreign body aspiration is a rare event, but it should be considered as a potential diagnosis in patients presenting with persistent symptoms or new complaints with a positive history of foreign body aspiration.
